# Early prediction of response to chemotherapy with Cetuximab in colorectal liver metastasis with contrast-enhanced ultrasound perfusion analysis

**DOI:** 10.1186/s12876-026-04902-6

**Published:** 2026-05-09

**Authors:** Najiao Tang, Peishan Guan, Daohui Yang, Longhui Zhang, Lewu Lin, Tingting Li, Aiqin Wu, Qing Lu

**Affiliations:** 1 Department of Ultrasound, Zhongshan Hospital (Xiamen), Fudan University, Xiamen, China; 2 Department of Ultrasound, Zhongshan Hospital, Institute of Ultrasound in Medicne and Engineering, Fudan University, Shanghai, China; 3 Xiamen Clinical Research Center for Cancer Therapy, Xiamen, China

**Keywords:** Prediction, Early response, Contrast media, Algorithms, Liver metastasis, Colorectal carcinoma

## Abstract

**Background:**

To evaluate the feasibility of contrast-enhanced ultrasound (CEUS) for early prediction of treatment response to chemotherapy combined with Cetuximab (Cet) in colorectal liver metastasis (CRLM).

**Methods:**

From June 2023 and October 2024, 139 consecutive patients with CRLM who underwent chemotherapy with Cet were sequentially allocated into a training cohort (*n* = 105) and a validation cohort (*n* = 34). CEUS examinations were conducted pre-treatment at baseline (week 0) and at weeks 2, 4, 6 and 8 post-treatment, with one target lesion consistently monitored throughout the therapeutic course. Perfusion parameters were derived using SonoLiver software. The reductions (Δ) and ratios of these parameters from baseline to each subsequent follow-up time point were compared between responders and non-responders.

**Results:**

According to mRECIST criteria, 83 patients (training cohort: *n* = 62, validation cohort: *n* = 21) and 56 patients (training cohort: *n* = 43, validation cohort: *n* = 13) were classified as responders and non-responders, respectively. In the training cohort, responders demonstrated significantly smaller tumor diameters compared to non-responders beginning at week 4 (4.6 ± 2.3 cm vs. 5.4 ± 2.8 cm, *p* = 0.027). From week 2 onward, the reductions and ratios of maximum intensity (IMAX) and area under curve (AUC) in responders were significantly higher compared to non-responders. At all time points (weeks 2, 4, and 6), the diagnostic performance of IMAX and AUC ratios was superior to that of ΔIMAX and ΔAUC. Furthermore, the AUROCs of IMAX and AUC ratios at week 4 were significantly higher than those at week 2 (Z = 3.531, *p* < 0.05; Z = 3.550, *p* < 0.05, respectively) and comparable to those at week 6 (Z = 1.596, *p* = 0.11; Z = 1.566, *p* = 0.12, respectively). In the validation cohort at week 4 post-treatment, the AUROCs of IMAX and AUC ratios were 0.896 and 0.905 (both *p* < 0.05), with corresponding accuracies of 85.3% and 85.3%.

**Conclusion:**

The ratios of AUC and IMAX at week 4 post-treatment may serve as reliable early predictors of treatment response to chemotherapy combined with Cetuximab in patients with CRLM.

## Background

Colorectal cancer (CRC) is one of the most prevalent malignancies globally, with approximately 30%-50% of CRC patients developing liver metastasis during their disease course [1]. Although surgical resection remains the only potentially curative treatment for colorectal liver metastasis (CRLM), it is not feasible in 75% CRLM cases due to extensive tumor burden or multifocal involvement. For these patients, FOLFOX (5-FU, leucoverin, and oxaliplatin) or FOLFIRI (5-FU, leucoverin, and irinotecan) are commonly used as first-line chemotherapy regimens [2],. The incorporation of Cetuximab (Cet), an epidermal growth factor receptor (EGFR)-targeted monoclonal antibody, in combination with conventional chemotherapy has expanded treatment options for unresectable CRLM [3].

According to the National Comprehensive Cancer Network (NCCN) Clinical Practice Guidelines for Colon Cancer (2021), FOLFOX plus Cet or FOLFIRI plus Cet are recommended as first-line standard regimens for CRLM, wherein chemotherapy forms the backbone of treatment, and Cetuximab confers an additional targeted effect [3]. However, this combination therapy elicits a response in only a subset of patients and is consistently associated with a high incidence of adverse events and considerable financial burden [4]. Consequently, the early identification of patients who are unlikely to respond is critical to prevent unnecessary toxic exposure and enable prompt modification of therapeutic strategy.

Traditionally, the modified Response Evaluation Criteria in Solid Tumors (mRECIST) has been the predominant approach for evaluating tumor response, emphasizing the assessment of viable tumor tissue characterized by enhancement on dynamic imaging techniques such as contrast-enhanced computed tomography (CECT) or dynamic contrast-enhanced magnetic resonance image (DCE-MRI) [5, 6]. However, mRECIST-based evaluations are generally conducted 6 to 8 weeks after initiation of treatment, equivalent to approximately 3–4 cycles of therapy, potentially delaying the acquisition of crucial response data [7]. Hence, there is an imperative need for a reliable and effective surrogate marker to facilitate early prediction of treatment response, enabling timely transition of non-responders to alternative therapeutic regimens.

Preclinical and clinical investigations have demonstrated that the additive effect of Cet, mediated through blockade of the EGFR signaling pathway and subsequent downregulation of downstream pro-angiogenic factors such as vascular endothelial growth factor (VEGF) and angiopoietins, results in reduced tumor blood volume and perfusion. These hemodynamic alterations manifest within hours to days following treatment initiation, substantially earlier than observable changes in tumor volume [8–12]. In light of these observations, functional imaging modalities have gained attention as promising tools for early prediction of therapeutic response to Cet-containing regimens by quantitatively assessing tumor perfusion dynamics. However, the optimal imaging time points and specific analytical methodologies remain undetermined [13–15]. Owing to the purely intravascular nature of the contrast agent SonoVue, contrast-enhanced ultrasound (CEUS) perfusion analysis provides a reliable assessment of blood volume and perfusion within solid lesions [16]. This suggests that CEUS may serve as a valuable tool for assessing and predicting treatment responses in malignant tumors. Moreover, CEUS is widely available, cost-effective compared with CT or MRI, and free of ionizing radiation, making it an attractive modality for repeated early treatment monitoring.

This study aimed to prospectively evaluate the feasibility and optimal timing of CEUS perfusion analysis for early prediction of treatment response in CRLM patients undergoing chemotherapy combined with Cet. This present article adheres to the STARD reporting checklist.

## Materials and methods

### Patients

**Fig. 1:**
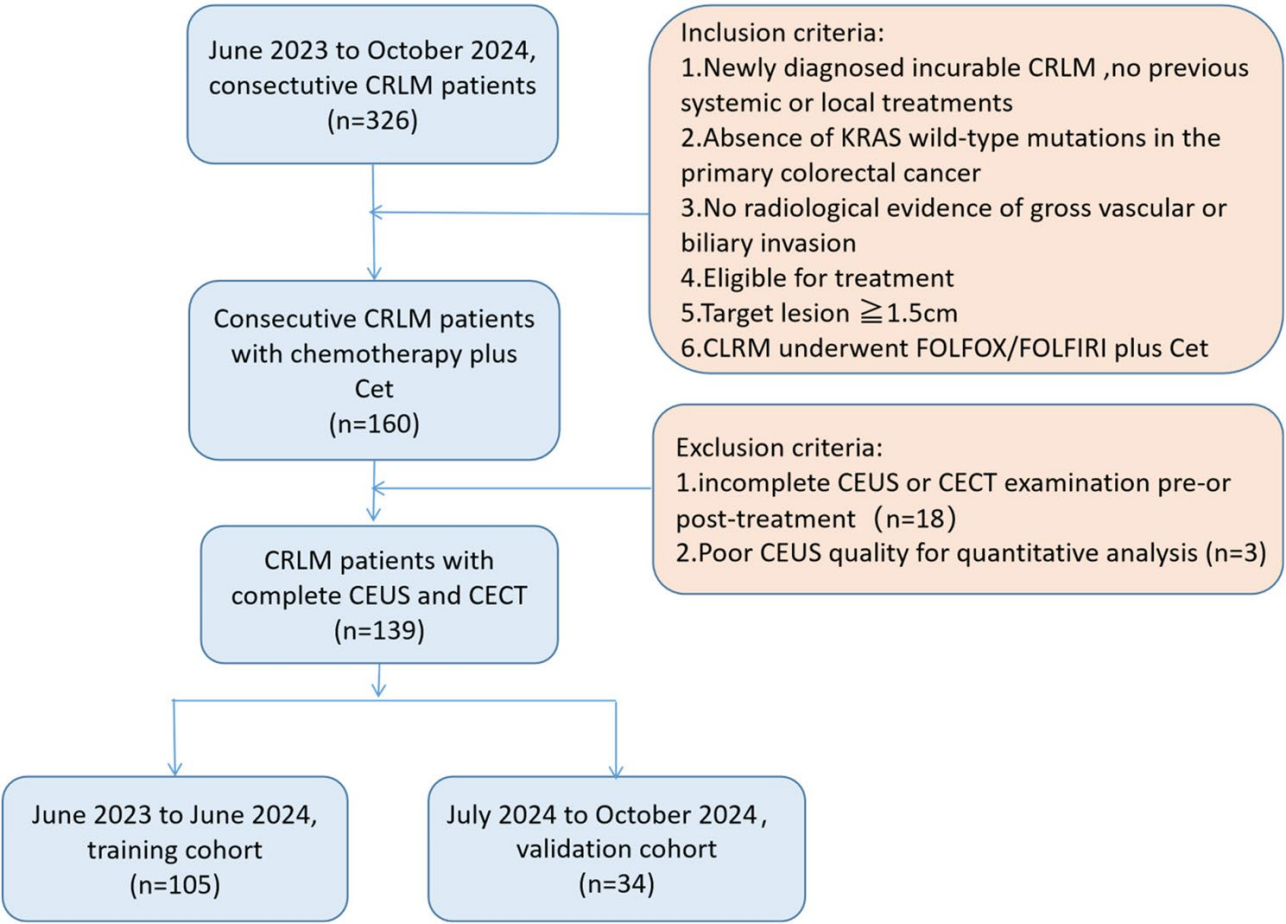
Flowchart of the inclusion of study subjects

The institutional review board granted approval for this prospective study (No. B2022-347R), and written informed consent was obtained from all participants. This study was conducted in accordance with the Declaration of Helsinki ( revised in 2013). From June 2023 to October 2024, a total of 326 consecutive patients with CRLM were enrolled for CEUS examinations at our institute. The inclusion criteria were defined as follows: (1) patients with newly diagnosed CRLM for whom curative treatments (e.g., radiofrequency ablation, resection, etc.) were not viable, and those without previous systemic or local treatments (e.g., chemotherapy, transarterial chemoembolization (TACE), etc.); (2) absence of KRAS mutations confirmed by pathological genetic analysis of the primary colorectal tumor; (3) no radiological evidence of gross vascular or biliary invasion, as assessed via CECT, MRI, or ultrasound; (4) adequate renal and liver function; (5) maximum diameter of target lesion ≥ 1.5 cm, along with an acoustic window suitable for stable CEUS data acquisition; (6) treatment regimens being either FOLFOX or FOLFIRI plus Cet, decided by clinicians based on a comprehensive and personalized treatment strategy tailored to the patient’s overall condition. The CEUS protocol stipulated examinations at week 0 (prior to treatment initiation) and at weeks 2, 4, 6, and 8 post-treatment. Exclusion criteria comprised: (1) incomplete CEUS examinations (missing one or more scheduled pre- or post-treatment examinations) or incomplete CECT examination (missing either pre-treatment or 8-week post-treatment imaging) (*n* = 18); (2) suboptimal CEUS image quality due to factors such as fragile breath-holding, ultrasound absorption due to deep tumor location or severe fatty liver (*n* = 3). Finally, 139 patients with CRLM were prospectively included and chronologically assigned to a training cohort (June 2023 to June 2024, *n* = 105) and a validation cohort (July 2024 to October 2024, *n* = 34) (Fig. 1).

### CEUS examinations

A radiologist with 16 years of experience in liver CEUS conducted all examinations using an Epiq 7 system (3.5 MHz convex-array probe; Philips Healthcare, Bothell, WA, USA). On gray-scale ultrasound, a target lesion was identified, and the imaging plane was optimally selected to simultaneously capture the largest section of the target lesion along with the adjacent liver parenchyma. Given that a single administration of contrast agent allows for the observation of only one lesion in CEUS examinations, for patients with multiple lesions, the criteria for selecting the target lesion were as follows: (1) the largest lesion; (2) a lesion within an acoustic window of sufficient quality; (3) a lesion adjacent to recognizable anatomical landmarks (e.g., gallbladder, hepatic vein, or portal vein) to facilitate consistent and precise localization across subsequent follow-ups; (4) a lesion that remained stable to minimize motion artifacts induced by respiration during quantitative analysis. The same target lesion was consistently re-evaluated in biweekly CEUS follow-up examinations. The maximum diameter of the lesion was measured using electronic calipers.

During CEUS examination, the contrast agent SonoVue (Bracco, Italy) was administered intravenously as a bolus (2.0mL), followed by a 5-mL flush of 0.9% sodium chloride. All CEUS examinations were performed using consistent technical settings:: depth 14 cm, gain 75%, mechanical index 0.06, dynamic range 70dB, temporal resolution 10 frames per second, with one focus positioned below the lesion. Patients were instructed to hold their breath gently, and the probe was held steady to prevent significant motion of the lesion in the ultrasound image. Each CEUS acquisition lasted at least three minutes, and cine loops were archived in DICOM format on a hard disk for subsequent offline quantitative analysis.

### Quantitative CEUS perfusion analysis

**Fig. 2:**
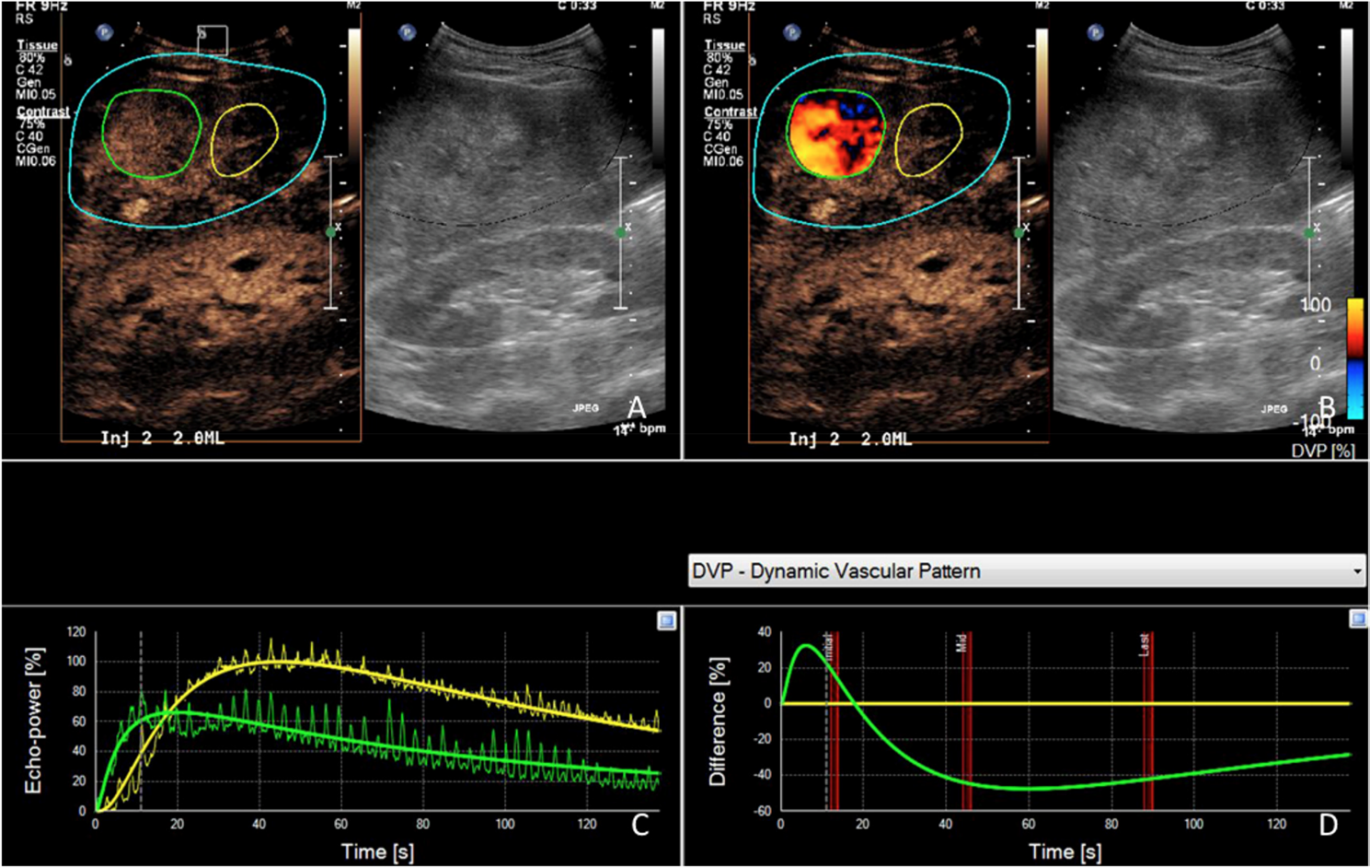
A 55-year-old man with colorectal liver metastasis (CRLM), target lesion measured 42 mm. SonoLiver screenshot of contrast-enhanced ultrasound image and dynamic perfusion image with motion compensation are shown. **A**, **B** Ultrasound image (**A**) and color-coded display of dynamic perfusion model diagram (**B**) show CRLM. Three regions of interest (ROIs) are drawn: area within the blue line is the motion compensation area, a reference ROI (yellow line), and an analysis ROI covering the whole lesion (green line). **C** Contrast agent dynamics in the reference area (yellow line) and analysis area (green line). Thin lines are the original dynamic perfusion curve, and thick lines are the perfusion curve after best-fitting analysis. **D** Time-to-intensity curve of the difference between original signals in the analysis ROI (green line) and reference signal averaged in the reference ROI

An additional radiologist, blinded to patients’ clinical information and with 10-year experience in CEUS interpretation, conducted CEUS perfusion analysis using SonoLiver software (Bracco Research SA, Geneva, Switzerland, and TomTec Imaging Systems GmbH, Unterschleissheim, Germany). The process details were as follows: (1) CEUS video clips in DICOM format were imported into the software database; (2) respiratory motion was automatically corrected using the motion compensation function integrated within the SonoLiver software; (3) regions of interest (ROIs) were defined: the analysis ROI was meticulously manually drawn to encompass the entire target lesion as much as possible, thereby representing the average perfusion of the entire lesion, regardless of internal enhancement defects or heterogeneity; a reference ROI was drawn in the adjacent liver parenchyma at the same depth as the analysis ROI, avoiding artifacts, large vessels, and the liver capsule. The shape and size of the analysis ROI varied considerably depending on the target lesion, whereas the reference ROI was round and ranged from 1 to 2 cm in diameter; (4) perfusion data processing: the time-intensity curve (TIC) was automatically generated based on signal intensity derived from echo-power values following log-compressed video images and was smoothed using a mathematical model tailored for bolus kinetics (Fig. 2). Analysis commenced at time 0 s, defined as the arrival of contrast agent in the hepatic artery. The quality of fit (QOF) was automatically calculated by the software to ascertain the congruence between the original echo-power signal and the refined perfusion model. In our study, a QOF ≥ 80% was required for each lesion to ensure perfusion analysis quality; those with QOF < 80% underwent repeated analysis, and those still failing to meet the criterion were excluded from the study; (5) five perfusion parameters were extracted from the TIC: (a) maximum intensity (IMAX), defined as the peak intensity of the tumor TIC divided by the intensity of the liver parenchyma × 100%; (b) area under curve (AUC), integration of intensity under the tumor TIC; (c) rise time (RT), time taken for the intensity to ascend from 10% to 100% of the peak on tumor TIC; (d) time-to-peak (TTP), time interval from contrast agent emergence to peak intensity on the tumor TIC; (e) mean transit time (MTT), time taken for the intensity to decline from peak to 50% of the peak value on the tumor TIC. The reduction and ratio of perfusion parameters between baseline (week 0) and different post-treatment time points were designated as ΔPar. (ΔPar.=Par. pre-treatment - Par. post-treatment) and ratio of Par. (ratio of Par. = ΔPar. / Par. pre-treatment), respectively.

### Endpoints and response evaluation criteria

In present study, the endpoint was established at the completion of 4 cycles (8 weeks after treatment initiation). Tumor response was evaluated using mRECIST as the standard reference at week 8, based on changes from baseline in the longest viable tumor diameter, defined as enhancement observed on CECT [5]. An independent panel of radiologists, blinded to clinical information and CEUS results, graded patients into four groups according to mRECIST: (1) complete response (CR), disappearance of any intratumoral arterial enhancement; (2) partial response (PR), a decrease of at least 30% in the sum of diameters of viable target lesions relative to baseline; (3) progressive disease (PD), an increase of at least 20% in the sum of diameters of viable target lesions; and (4) stable disease (SD), encompassing cases not meeting the criteria for CR, PR, or PD [5, 17]. For the purposes of this analysis, patients achieving CR or PR were classified as responders, whereas those with SD or PD were classified as non-responders.

### Statistical analysis

Statistical analyses were performed using SPSS v.25.0 (SPSS Inc., Chicago, IL, USA). For each patient, changes (reduction and ratio) in CEUS perfusion parameters from baseline (week 0) to each subsequent post-treatment time point were quantified. The intra-class correlation coefficient (ICC) was used to evaluate the consistency of intra-reader interpretations, with agreement levels defined as follows: perfect when ICC ≥ 0.75, moderate when 0.4 ≤ ICC < 0.75, and poor when ICC < 0.4. Differences in tumor diameter and perfusion parameters changes between responders and non-responders were assessed using independent *t* test. A two-tailed *p-*value of less than 0.05 was considered statistically significant. Correlations between significant perfusion parameters and mRECIST grades were assessed via Spearman’s correlation coefficient analysis, interpreted as follows: correlation coefficient *r* > 0.7 indicates a strong correlation, 0.4 ≤ *r* ≤ 0.7 indicates a moderate correlation, and *r* < 0.4 indicates a weak correlation. The performance of perfusion parameters in early prediction of treatment response was evaluated by analyzing the area under the receiver operating characteristic curve (AUROC). Accuracy, sensitivity, specificity, positive predictive value (PPV), and negative predictive value (NPV) were calculated based on cut-off values that maximize the sum of sensitivity and specificity.

## Results

### Patient characteristics

The flowchart illustrating study participant recruitment is depicted in Fig. 1. A total of 139 patients (105 in the training cohort, 34 in the validation cohort) were enrolled in this study. Clinical characteristics are summarized in Table 1. Statistical analysis revealed that all clinical features, including patient number, gender, age, primary tumor location, tumor sidedness and pathology, number of tumors, tumor size, and levels of carcinoembryonic antigen (CEA) and carbohydrate antigen 19 − 9 (CA19-9), were not statistically different bertween responders and non-responders (*p >* 0.05 for all).

**Table 1:** Baseline characteristics of study subjects

Characteristic	Training cohort (*n* = 105)			Validation cohort (*n* = 34)		
	Responders	Non-responders	*P* value	Responders	Non-responders	*P* value
Case number (count)	62	43		21	13	
Male/female (count)	41/21	29/14	0.888	15/6	8/5	0.549
Age (y, mean ± SD)	59.7 ± 7.8	60.1 ± 7.9	0.813	61.8 ± 7.4	58.6 ± 9.8	0.295
Primary tumor location			0.496			0.434
Colon (count, %)	47 (75.8%)	35 (81.4%)		17 (81.0%)	9 (69.2%)	
Rectum (count, %)	15 (24.2%)	8 (18.6%)		4 (19.0%)	4 (30.8%)	
Primary tumor sidedness			0.107			0.384
Left-sided (including rectum) (count, %)	57 (91.9%)	35 (81.4%)		19 (90.5%)	10 (76.9%)	
Right-sided (including transverse colon) (count, %)	5 (8.1%)	8 (18.6%)		2 (9.5%)	3 (23.1%)	
Primary tumor pathology			0.841			0.961
Mucinous carcinoma (count, %)	14 (22.6%)	9 (20.9%)		5 (23.8%)	3 (23.1%)	
Non-mucinous carcinoma (count, %)	48 (77.4%)	34 (79.1%)		16 (76.2%)	10 (76.9%)	
Number of tumors (median, range)	11.3 (5–19)	9.6 (4–22)	0.416	10.3 (4–18)	10.8 (4–20)	0.094
Tumor size (cm, mean ± SD)	5.2 ± 2.6	4.8 ± 2.2	0.692	4.9 ± 2.1	5.5 ± 1.9	0.613
Biomarkers						
CEA (ng/ml)	1355 ± 956	1474 ± 1027	0.583	1523 ± 661	1420 ± 886	0.186
CA19-9 (U/ml)	574 ± 263	526 ± 301	0.772	508 ± 219	449 ± 269	0.384

*CEA* Carcino-embryonic antigen, *CA19-9* Carbohydrate antigen 19 − 9

All eligible patients received either FOLFOX (5-fluorouracil/ leucovorin/ oxaliplatin) plus cetuximab (Cet) (*n* = 103) or FOLFIRI (5-fluorouracil/ leucovorin/ irinotecan) plus cetuximab (Cet) (*n* = 36) as their standard treatment protocol, determined by clinicians based on a comprehensive and personalized treatment strategy tailored to the patient’s overall condition. According to mRECIST criteria assessed at 8 weeks post-treatment, 83 patients (62 in the training cohort, 21 in the validation cohort) were classified as responders (83 PR and 0 CR), while 56 patients (43 in the training cohort, 13 in the validation cohort) were classified as non-responders (39 SD and 17 PD) (*p* = 0.779). Among the 83 responders, 60 received FOLFOX + Cet and 23 received FOLFIRI + Cet. Similarly, among the 56 non-responders, 43 received FOLFOX + Cet and 13 received FOLFIRI + Cet (*p* = 0.553).

### Intra-reader agreement analysis

To rigorously assess intra-reader agreement, a random subset of 30 subjects was selected for ICC analysis. CEUS perfusion parameters at distinct time-points were quantitatively re-analyzed by the same radiologist one week after the initial assessment. Results demonstratied perfect agreement with the initial measurements, yielding ICC values of 0.834, 0.801, 0.791, 0.809, and 0.790 at weeks 0, 2, 4, 6, and 8, respectively.

### Efficacy of perfusion parameters in early prediction of response in the training cohort

Maximum tumor diameters of responders and non-responders were compared at distinct time points. Prior to treatment initiation (week 0), tumor size was comparable between responders and non-responders (5.2 ± 2.6 cm vs. 4.8 ± 2.2 cm, *p* = 0.692). At week 2, no signigicant difference in tumor size was observed between responders and non-responders (5.2 ± 2.5 cm vs. 5.0 ± 2.4 cm, *p* = 0.784). However, at weeks 4, 6, and 8, tumor diameters in responders were all significantly smaller than those in non-responders (week 4: 4.6 ± 2.3 cm vs. 5.4 ± 2.8 cm, *p* = 0.027; week 6: 4.4 ± 1.9 cm vs. 5.5 ± 3.0 cm, *p* = 0.002; week 8: 4.0 ± 1.8 cm vs. 6.1 ± 3.2 cm, *p* < 0.001).

Comparative analyses of reductions and ratios of CEUS perfusion parameters between responders and non-responders in the training cohort at distinct time points are summarized in Tables 2 and 3, respectively. At all time points after treatment initiation, ΔIMAX and ΔAUC, as well as the corresponding ratios of IMAX and AUC, were significantly higher in responders compared to non-responders. In contrast, no significant differences were observed in reductions or ratios of RT, TTP, or MTT between the two groups. Notably, as early as week 2, significant differences were observed between responders and non-responders in both reductions and ratios of IMAX and AUC.

**Table 2:** Comparisons of reductions in CEUS perfusion parameters at different time points between responders and non-responders in the training cohort

Change of Parameter	Week 2	Week 4	Week 6	Week 8
ΔIMAX				
Responders	38.26 ± 40.49	55.97 ± 47.17	67.00 ± 53.41	73.72 ± 56.23
Non-responders	7.36 ± 8.22	8.90 ± 10.90	8.49 ± 14.05	8.49 ± 16.43
* P* value	< 0. 001	< 0. 001	< 0. 001	< 0. 001
ΔAUC/100				
Responders	26.95 ± 27.81	37.00 ± 29.94	42.96 ± 33.89	46.93 ± 36.03
Non-responders	3.66 ± 6.67	4.67 ± 8.64	5.29 ± 9.75	6.04 ± 11.67
* P* value	< 0. 001	< 0. 001	< 0. 001	< 0. 001
ΔRT (s)				
Responders	-0.25 ± 3.88	-0.03 ± 4.05	-0.32 ± 5.70	-0.25 ± 6.22
Non-responders	0.41 ± 3.01	0.03 ± 3.48	0.54 ± 4.31	0.57 ± 4.98
* P* value	0.350	0.940	0.403	0.472
ΔTTP (s)				
Responders	-0.19 ± 4.26	0.05 ± 4.49	0.16 ± 5.89	-0.27 ± 6.77
Non-responders	0.48 ± 3.36	0.28 ± 3.82	0.58 ± 4.68	0.61 ± 5.21
* P* value	0.397	0.787	0.702	0.476
ΔMTT (s)				
Responders	-10.44 ± 88.78	-23.49 ± 96.75	-37.59 ± 128.57	-13.58 ± 132.92
Non-responders	-6.06 ± 94.51	-20.01 ± 133.51	-7.28 ± 170.85	-6.51 ± 203.05
* P* value	0.809	0.877	0.302	0.830

*IMAX* Maximum intensity, *AUC* Area under curve, *RT* Rise time, *TTP* Time to peak, *MTT* Mean transit time

**Table 3:** Comparisons of ratios of CEUS perfusion parameters at different time points between responders and non-responders in the training cohort

Change of Parameter	Week 2	Week 4	Week 6	Week 8
IMAX ratio				
Responders	0.31 ± 0.17	0.48 ± 0.15	0.59 ± 0.15	0.66 ± 0.15
Non-responders	0.10 ± 0.10	0.12 ± 0.16	0.10 ± 0.25	0.09 ± 0.29
* P* value	< 0. 001	< 0. 001	< 0. 001	< 0. 001
AUC ratio				
Responders	0.33 ± 0.20	0.50 ± 0.18	0.59 ± 0.19	0.66 ± 0.19
Non-responders	0.08 ± 0.14	0.11 ± 0.18	0.12 ± 0.22	0.13 ± 0.26
* P* value	< 0. 001	< 0. 001	< 0. 001	< 0. 001
RT ratio				
Responders	-0.07 ± 0.27	-0.06 ± 0.30	-0.10 ± 0.41	-0.10 ± 0.44
Non-responders	0.01 ± 0.25	-0.05 ± 0.33	-0.03 ± 0.40	-0.03 ± 0.45
* P* value	0.169	0.864	0.371	0.436
TTP ratio				
Responders	-0.07 ± 0.27	-0.05 ± 0.29	-0.05 ± 0.39	-0.10 ± 0.45
Non-responders	-0.01 ± 0.26	-0.04 ± 0.34	-0.03 ± 0.42	-0.04 ± 0.45
* P* value	0.229	0.796	0.731	0.493
MTT ratio				
Responders	-0.42 ± 1.82	-0.57 ± 2.50	-0.81 ± 2.94	-0.74 ± 3.30
Non-responders	-0.15 ± 0.46	-0.23 ± 0.84	-0.27 ± 0.84	-0.21 ± 1.01
* P* value	0.345	0.389	0.248	0.308

*IMAX* Maximum intensity, *AUC* Area under curve, *RT* Rise time, *TTP* Time to peak, *MTT* Mean transit time

Correlation coefficients between perfusion parameters at various post-treatment time points and mRECIST grades at week 8 are presented in Table 4. Specifically, ΔIMAX, ΔAUC, and ratios of IMAX and AUC at week 2 demonstrated moderate correlations with mRECIST. Furthermore, ΔIMAX, ratios of IMAX and AUC at week 4, and ΔIMAX, ΔAUC, and ratios of IMAX and AUC at weeks 6 and 8 exhibited strong correlations with mRECIST.

**Table 4:** Correlation coefficients between perfusion parameters and mRECIST grades in the training cohort

Time	Reduction of parameters		Ratio of parameters	
	ΔIMAX	ΔAUC	IMAX ratio	AUC ratio
Week 2	*r* = 0.591	*r* = 0.583	*r* = 0.627	*r* = 0.604
Week 4	*r* = 0.717	*r* = 0.686	*r* = 0.776	*r* = 0.738
Week 6	*r* = 0.750	*r* = 0.713	*r* = 0.805	*r* = 0.763
Week 8	*r* = 0.760	*r* = 0.710	*r* = 0.818	*r* = 0.757

*IMAX* Maximum intensity, *AUC* Area under curve

**Fig. 3:**
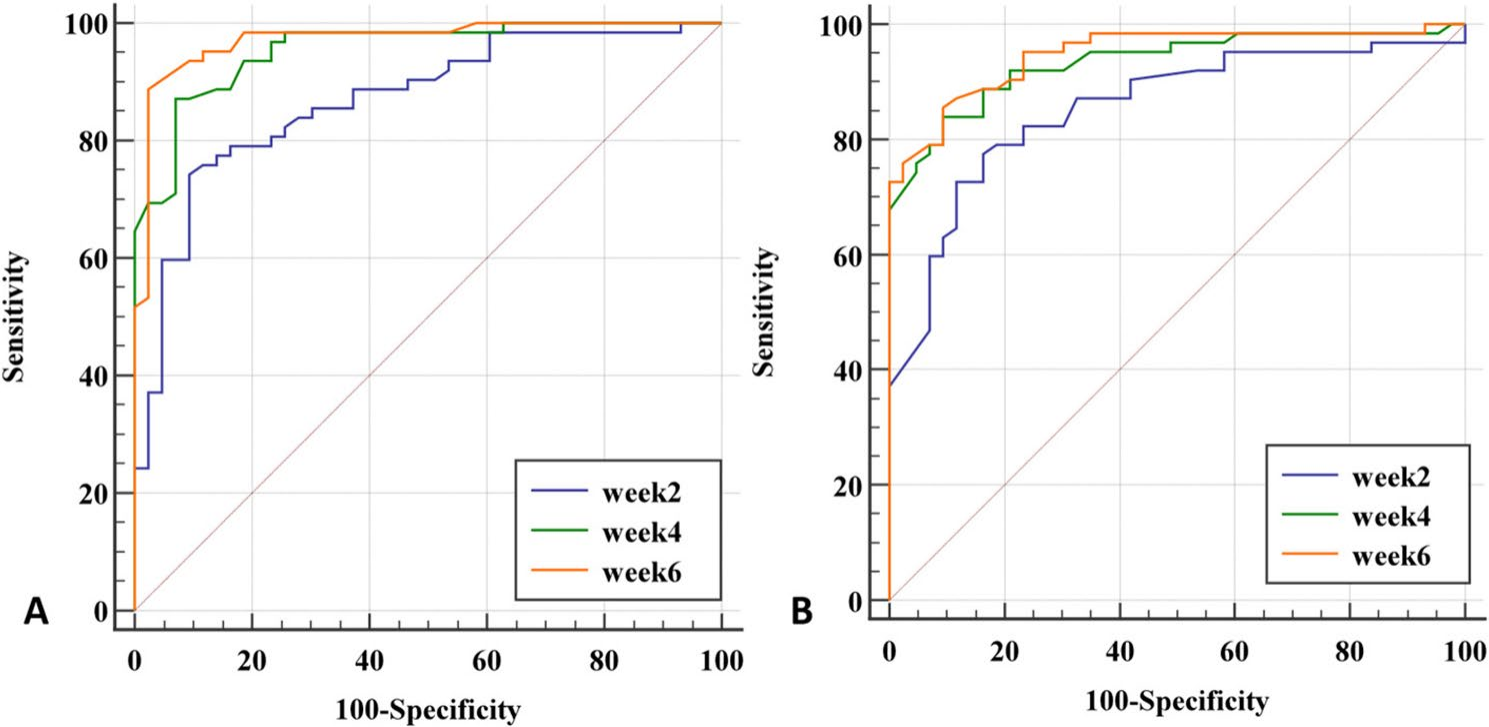
AUROCs of IMAX ratio (**A**) and AUC ratio (**B**) at weeks 2, 4 and 6 in the training cohort

Table 5 summarizes the diagnostic performance of perfusion parameters in early prediction of response. The diagnostic efficacy of IMAX and AUC ratios was superior to that of ΔIMAX and ΔAUC at all time points (weeks 2, 4, and 6). Furthermore, pairwise comparison of diagnostic efficacy showed that AUROCs of IMAX ratio and AUC ratio at week 4 were signigicantly higher than those at week 2 (Z = 3.531, *p* < 0.05; Z = 3.550, *p* < 0.05) and comparable to those at week 6 (Z = 1.596, *p* = 0.11; Z = 1.566, *p* = 0.12) (Fig. 3).

**Table 5:** Efficacy of perfusion parameters at weeks 2, 4, and 6 in predicting mRECIST response in the training cohort

	AUROC	Youden Index	Cut-off	Accuracy	Sensitivity	Specificity	PPV	NPV
ΔIMAX (week 2)	0.847 (0.774–0.920)	0.601	14.84	78.1%	69.4%	90.7%	91.5%	67.2%
ΔAUC/100(week 2)	0.842 (0.768–0.917)	0.561	10.06	76.2%	67.6%	88.4%	89.4%	65.5%
IMAX ratio(week 2)	0.868 (0.799–0.937)	0.649	0.185	81.0%	74.2%	90.7%	92.0%	70.9%
AUC ratio (week 2)	0.854 (0.781–0.928)	0.611	0.185	80.0%	77.4%	83.7%	87.3%	72.0%
ΔIMAX (week 4)	0.921 (0.871–0.970)	0.717	16.17	86.7%	90.3%	81.4%	87.5%	85.4%
ΔAUC/100(week 4)	0.903 (0.846–0.960)	0.681	14.09	82.9%	77.4%	90.7%	92.3%	73.6%
IMAX ratio(week 4)	0.955 (0.920–0.990)	0.801	0.305	89.5%	87.1%	93.0%	94.7%	83.3%
AUC ratio(week 4)	0.933 (0.886–0.980)	0.746	0.355	86.7%	83.9%	90.7%	92.9%	79.6%
ΔIMAX(week 6)	0.940 (0.899–0.982)	0.743	16.82	88.6%	95.2%	79.1%	86.8%	91.9%
ΔAUC/100(week 6)	0.919 (0.867–0.970)	0.699	14.94	84.8%	83.9%	86.0%	89.7%	78.7%
IMAX ratio(week 6)	0.972 (0.943-1.000)	0.864	0.395	92.4%	88.7%	97.7%	98.2%	85.7%
AUC ratio(week 6)	0.948 (0.907–0.988)	0.762	0.415	87.6%	85.5%	90.7%	93.0%	81.3%

*IMAX* Maximum intensity, *AUC* Area under curve, *AUROC* Area under the receiver operating characteristic curve, *PPV* Positive predictive value, *NPV* Negative predictive value

### Efficacy of perfusion parameters in early prediction of response in the validation cohort

**Fig. 4:**
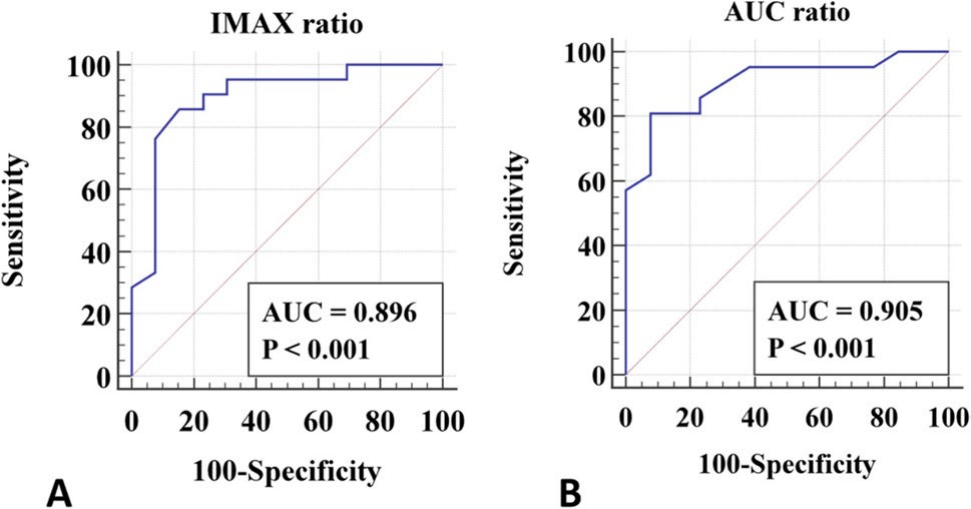
AUROCs of IMAX ratio (**A**) and AUC ratio (**B**) at week 4 in the validation cohort

In the validation cohort, 21 and 13 patients were categorized as responders and non-responders, respectively. Before treatment initiation (week 0), tumor diameters were comparable between the two groups (4.9 ± 2.1 cm vs. 5.5 ± 1.9 cm, *p* = 0.613). At week 4, tumor diameters in responders were slightly smaller than those in non-responders (4.7 ± 1.7 cm vs. 5.8 ± 1.7 cm, *p* = 0.716). At the same time point, the AUROCs of IMAX ratio and AUC ratio at week 4 were 0.896 (95% CI: 0.742–0.974) and 0.905 (95% CI: 0.754–0.978), respectively, in predicting responders (*p* < 0.05 for both) (Fig. 4), with sensitivity, specificity, and accuracy of 85.7%, 84.6%, and 85.3% for IMAX ratio and 81.0%, 92.3%, and 85.3% for AUC ratio.

## Discussion

CEUS perfusion parameter analysis is recognized as an effective indicator of dynamic vascular alterations in tumors following treatment, owing to its capacity to directly visualize blood flow changes. In the present study, although the difference in tumor diameters between responders and non-responders in the training cohort reached statistical significance at week 4, no such difference was observed in the validation cohort at the corresponding time point. Notably, as early as the first treatment session (week 2), the reductions and ratios of IMAX and AUC in responders were significantly greater compared to non-responders. These findings suggest that changes in IMAX and AUC may represent promising surrogate imaging biomarkers for the early prediction of treatment response, preceding notable tumor volume shrinkage. This observation aligns with prior investigations utilizing various functional imaging modalities for early assessment of treatment response in CRC and CRLM. Walker et al. reported that DCE-US could detect reduced tumor vascularity in responders as early as 1–2 weeks after treatment initiation [13]. Similarly, Lastoria et al. demonstrated that PET/CT-assessed response after a single treatment cycle (week 2) held significant predictive value for long-term outcomes in CRLM patients receiving preoperative therapy [18]. These collective results indicate that alterations in tumor perfusion may serve as an early indicator of treatment efficacy in CRLM managed with chemotherapy and anti-angiogenic agents, and can be accurately quantified through functional imaging modalities, including CEUS perfusion parameters [19, 20].

CEUS enables the detection of treatment response prior to observable tumor volume reduction, a phenomenon that may be ascribed to several underlying biological mechanisms contributing to the dissociation between functional perfusion changes and morphological alterations. Firstly, vascular normalization. Cetuximab, by inhibiting EGFR downstream pro-angiogenic factors such as VEGF and angiopoietins, can induce normalization of aberrant tumor vasculature. This leads to a measurable decrease in CEUS perfusion parameters (IMAX and AUC) before tumor shrinkage becomes detectable [21, 22]. Secondly, extensive tumor necrosis. Effective treatment can cause rapid and widespread necrosis, but necrotic tissue is not always absorbed immediately; the tumor may therefore remain unchanged in size while CEUS already shows perfusion defects in the necrotic areas [23]. Thirdly, tissue fibrosis. In some patients, early fibrotic changes may develop around tumors following treatment. Fibrotic tissue can maintain tumor volume while being avascular, leading to reduced CEUS perfusion without corresponding size reduction [24].These mechanisms provide insight into why CEUS can serve as an early indicator of treatment response and illustrate the complementary value of functional and morphological assessment in evaluating therapeutic efficacy.

In the present study, a comparison of the AUROCs for IMAX and AUC ratios across various time points revealed that parameters at week 4 and week 6 exhibited comparable predictive performance for treatment response, and both significantly outperformed those at week 2. These findings suggest that week 4 may serve as the optimal time point for early prediction of radiologic response based on mRECIST criteria assessed at week 8. Using cut-off values of > 0.305 and > 0.355 for IMAX and AUC ratios, respectively, both parameters achieved relatively high accuracy, sensitivity, and specificity within both the training and validation cohorts. These results align with previous reports by Schirin-Sokhan et al., who demonstrated the utility of CEUS for early response prediction following anti-angiogenic therapy in CRLM after the second treatment cycle (week 4) [25]. Similarly, Tranquart et al. indicated that CEUS perfusion parameters measured at baseline and during follow-up on day 15 or day 30 are most relevant for identifying patients with less effective treatment [26]. In accordance with these observations, Amadori et al. reported that CEUS could detect and quantify dynamic alterations in tumor vascularity as early as 15 days after therapy [27].Thus, the collective evidence suggests that for CRLM patients undergoing chemotherapy combined with Cet, CEUS perfusion analysis as early as week 4 may facilitate early prediction of treatment response. Notably, this early functional assessment at week 4 carries substantial clinical relevance, particularly when integrated with conventional morphological response criteria such as early tumor shrinkage (ETS) evaluated at week 8.

Early tumor shrinkage (ETS) assessed at week 8 has been established as a prognostic indicator in patients with CRLM receiving anti-EGFR antibody-combined chemotherapy [28]. However, similar to mRECIST, ETS evaluation is constrained by the inherent delay in therapeutic response assessment. Our findings demonstrate that CEUS perfusion parameters at week 4 achieve predictive performance comparable to that at week 6 (AUROC > 0.9), and significantly outperform the predictive capability observed at week 2. This supports the adoption of week 4 as an optimal time point for functional response evaluation. Notably, this approach affords a 4-week advancement compared to conventional ETS assessment at week 8. Therefore, CEUS at week 4 may serve as a functional imaging-based early warning system that complements ETS, enabling the identification of patients unlikely to benefit from ETS at week 8 and providing a critical opportunity for timely treatment adaptation. In clinical practice, for patients classified as CEUS non-responders at week 4, clinicians could consider earlier CECT evaluation (e.g., at week 6) and multidisciplinary consultation to facilitate earlier treatment modification. Such a proactive clinical strategy may mitigate unnecessary exposure to treatment-related toxicities, reduce healthcare costs, and preserve functional status for subsequent lines of therapies. Conversely, patients demonstrating CEUS response at week 4 may continue the current therapeutic regimen with standard ETS surveillance at week 8.

Additionally, our results indicated that at identical time points (week 2, 4, and 6), the diagnostic performance of IMAX and AUC ratios exceeded that of ΔIMAX and ΔAUC. This superior performance may be partly explained by inter-individual vascular heterogeneity, which contributes to a considerable variation in pre-treatment IMAX and AUC values among CRLM patients. Furthermore, physiological factors such as heart rate and cardiac function may further influence absolute IMAX and AUC values across different individuals. Therefore, during perfusion parameter analysis, the normalization of target lesion measurements can help mitigate certain sources of variability. Such normalization, as implemented in this study through the computation of perfusion value ratios (i.e., ratios of pre- to post-treatment tumor measurements), serves to reduce inter-subject discrepancies and enhance comparability [29, 30].

Our study has several limitations. First, the inclusion criteria were defined based on KRAS wild-type status rather than the currently recommended all-RAS wild-type status (including both KRAS and NRAS), necessitating future validation in all-RAS wild-type populations. Second, a substantial imbalance in tumor sidedness (87.1% left-sided) precluded a meaningful subgroup analysis; thus, our findings are primarily generalizable to left-sided, KRAS wild-type CRLM. Third, the relatively limited sample size necessitates further validation in larger multi-center trials. Fourth, the assessment of tumor perfusion based on a single two-dimensional section may introduce bias due to intratumoral vascular heterogeneity, a limitation that may be mitigated in future studies through the use of three-dimensional CEUS. Fifth, in cases of mixed treatment responses, comparative evaluation with CECT/MRI may provide a more comprehensive assessment beyond dimensional changes alone. Sixth, although intra-reader agreement was excellent (ICC: 0.790–0.834), we did not assess inter-reader reliability, and quantitative CEUS analysis is operator-dependent. Future studies should include inter-reader assessments and structured training programs. Finally, the relatively short follow-up duration impeded correlation analyses with progression-free survival or overall survival, which should be addressed in subsequent investigations.

## Conclusion

In conclusion, for patients with CRLM receiving chemotherapy in combination with Cet, AUC and IMAX at week 4 may serve as robust early predictors of treatment response evaluated according to mRECIST criteria at week 8. Furthermore, the ratios of IMAX and AUC demonstrate superior predictive performance compared to their corresponding reduction values in early treatment assessment.

## Data Availability

No datasets were generated or analysed during the current study.

## Abbreviations

CRC: Colorectal cancer
CRLM: Colorectal liver metastasis
FOLFOX: 5-FU, leucoverin and oxaliplatin
FOLFIRI: 5-FU, leucoverin and irinotecan
Cet: Cetuximab
EGFR: Epidermal growth factor receptor
mRECIST: The modified Response Evaluation Criteria in Solid Tumors
CECT: Contrast-enhanced computed tomography
DCE-MRI: Dynamic contrast-enhanced magnetic resonance image
CEUS: Contrast-enhanced ultrasound
ROI: The region of interest
TIC: The time-intensity curve
QOF: The quality of fit
IMAX: Maximum intensity
AUC: Area under curve
RT: Rise time
TTP: Time-to-peak
MTT: Mean transit time
CR: Complete response
PR: Partial response
PD: Progressive disease
SD: Stable disease
ICC: Intra-class correlation coefficient
AUROC: Area under the receiver operating characteristic curve
PPV: Positive predictive value
NPV: Negative predictive value
CEA: Carcinoembryonic antigen
CA19-9: Carbohydrate antigen 19 − 9
